# Temporary Teeth

**Published:** 1887-09

**Authors:** F. Beabody


### THE DENTAL REGISTER.

VOL. XLI.] SEPTEMBER, 1887. [No. 9.

### COMMUNICATIONS.

### The Temporary Teeth.

BY F. BEABODY, D.D.S.

Read before the Kentucky State Dental Society, June, 1887.

It is not the province of this paper to enter into the histology of the temporary teeth further than to state that the germs are found within the radius of the alveoli early in fœtal life; that the first calcific deposit is at or near that portion of the tooth subsequently occupied by the apex of the root, and that all continued calcification, both of crown and root, is at the expense of the pulp from which it is formed, which diminishes in proportion as the calcification advances, until it is reduced to the normal size, as found in the completed tooth. The pulp of the temporary tooth is not as sensitive as that found in the permanent, nor is the tooth as dense.

Its structure is more vascular and consequently admits of a greater degree of circulation.

The pulp is larger in proportion to the size of the tooth in the deciduous set, comes nearer the surface, and is more readily exposed by decay.

The temporary incisors come through the gums at a period of from three to six months after birth, though the generic law is often violated in the period and succession of their coming. Occasionally they are found projecting through the gums at birth. Again, one or two years may elapse before any of the teeth make their appearance.

The lateral incisors usually follow the centrals from one to three months later.

It is unusual for the centrals or laterals to cause any irritation in their eruption if they come in the regular course of time and succession, while it is seldom the case that the temporary teeth which come later do not cause more or less disturbance of the general system.

Occasionally a child will erupt all of its teeth with none of the symptoms usually attending this process.

It is the popular belief that many of the disorders common to infantile life arise directly from dentition. On this subject there is a diversity of opinion.

Formerly it was the accepted belief that much of the fever, restlessness, diarrhœa, and general irritability of the child during the period of dentition was due to the teeth. More recently it is held by many physicians that the teeth are in no way responsible for this pathological condition, that their eruption is purely physiological, and that the cause of irritation must be sought elsewhere.

That the eruption of the teeth, both temporary and permanent, is physiological can not be questioned, but many physiological efforts are attended by pathological results, and to say that the irruption of the temporary teeth causes no more disturbance than the growth of any of the other bones, the hair, or nails, is falling into the error which is too common in all who, with more enthusiasm than discretion, accept new theories—of going to extremes at the expense of facts.

Theories are constantly changing, those of to-day giving place to the newer and more acceptable one of to-morrow, but facts are incontrovertible, and must ever remain as proofs.

No one who has ever had the care of children can for one moment doubt that the teeth are the direct cause of many of their ailments, and when we consider how delicate and nicely balanced is the nervous organization of the child, it is hard to understand how physicians can say that the calcified crown of a tooth with a still uncalcified pulp pressing on a sensitive, highly organized gum, can fail to receive the sympathy of the nerves of other and more remote organs, as the stomach and bowels.

As before stated the eight anterior teeth, when coming in their regular course, and at their normal time, seldom cause irritation, that is where the child is naturally fed from its mother's milk, and that milk agrees with it. Children brought up on the bottle or fed artificially on any of the so-called improved or patented "food for infants," now so commonly in use, can not be expected to develop naturally, and must be more subject to the various diseases that the fragile organism in its struggle for more mature existence has to contend against. Why, to look upon an infant in its helplessness, and to note how entirely dependent it is for its future life on the care and wisdom bestowed on it by those who have it in charge, and the various accidents it is liable to, it is only a wonder that so many ever do arrive at maturity.

After the eruption of the eight anterior teeth, from six to nine months after birth, the child's food is generally changed, or in addition to its mother's milk (if it is so fortunate as to be fed in that way) other food is given to aid in supporting life, and to save the continued drain on the mother; and to this is attributed, by those holding the view that the teeth are not disturbing elements, the functional disturbances so common at this time of life, and until the teeth have all come through the gums, and that most if not all of the derangements of the child's health and comfort are owing to the irritation produced by the food received into the child's stomach, not yet strong enough to properly digest it.

Undoubtedly, this view of the case has some strong points in its support, but that the teeth are factors can not be denied.

The heat and inflammation of the swollen gums, produced by the pressure of the approaching tooth, extend throughout the mouth, and it is not unreasonable to suppose that the mucous membrane lining the stomach and intestines, which is continuous, is from sympathy in a susceptible and irritable condition from this cause, as much as from any food (which would naturally be of a simple character) which the child would have taken into its stomach. It is also reasonable to suppose that in this irritable condition the stomach would receive as an irritant food with which at other times it would harmonize and assimilate.

When a tooth is coming through the gums, say a molar or cuspid, the resistance is very great. Restlessness, nervousness, pain, general irritability, the fingers of the child frequently in the mouth, the excited condition of the salivary glands, shown in the profuse and constant flow of saliva, the relief given by pressure, all go to show that the tooth is the disturbing cause. If this is pressing hard, the gum over the point of the tooth is white from tension. A free incision by the lancet will generally produce relief; at times an almost instantaneous change will take place, and the fretfulness of the child pass away.

A single case in point will illustrate thousands of similar ones.

A child of twelve months, who showed evident signs of teething, being restless, feverish, irritable—so cross that nothing would appease it, so petulant that it could not bear to be spoken to, or even looked at; cries of pain every instant giving certain indications that the teeth were the cause of the trouble, was, after all topical applications had proved useless, taken from its bed at one o'clock in the morning, and the gum over the tooth freely opened. In five minutes after this trivial operation (so much dreaded by some), the child was sitting up in bed with its arms around its mother's neck, laughing, crowing, and in such a glee it took an hour for it to quiet down and go to sleep. What a change, and how quickly brought about; and yet some physicians will not use the lancet on a child's gums. Was this child suffering from irritating food? Was its pain caused by indigestion? Beyond doubt the cause was a local one, though its effects were general.

Formerly the lancet was in universal demand when the swollen gums indicated an approaching tooth. To-day many physicians appear to dread its use on a child's gums as though the operation was a formidable one, and it is only resorted to when all other means fail to relieve the pain.

Extensive inquiry among medical men who are opposed to the use of the lancet on infants when it is indicated has elicited but two reasons for withholding it: one the fear of hemorrhage, the other the cicatrix left from the wound which retards the eruption of the tooth. Neither of these reasons has any weight, for if the gum is properly lanced, care being taken not to go inside of the tooth, no hemorrhage will follow, nor will the gum be thickened over the cutting edge or grinding surface of the coming tooth, if instead of making the opening directly over it, it is made a little to the outer side; this will relieve the tension on the gum, and cause relaxation of the nervous strain on the system. Should the gum be lanced directly over the cutting or grinding surface of the tooth which is nearly ready to come through, the quick following of the tooth will prevent the wound from closing. Even on the cutting edges of the incisors and points of the cuspids a crucial incision is better than the direct line, and on molars it is absolutely necessary.

It has been suggested that it is not as much the pressure of the crown of the tooth on the gum as it is the backward pressure of the crown made by the resisting gum on the uncalcified pulp which causes the troubles of teething. This will hardly hold good, for even at times of the greatest irritation, pressure on the gums and rubbing will cause a momentary relief and seems gratifying to the child.

Topical applications to the gums will sometimes cause relaxation from pain. Among those in use the crystals of bromide of potash is perhaps one of the best, by rubbing the gums gently with a piece of the bromide large enough to handle. The benefit of rubbing is combined with the anæsthetic property of the bromide, and the benefit is sometimes marked. The warm bath, anything that will relax the system is beneficial.

Let me ask why it is that the physician, instead of the dentist is called in when a child is in pain from its teeth? Is it because the dentist is not supposed to know anything about the teeth and their relations to the surrounding tissues at such times? Or is it the fact that they are not generally familiar with the proper treatment of the teeth of infants? How many of you have been called in to attend to a case of teething and how often during your practice have you been called?

In a practice of thirty-five years your Committee can not recall more than half a dozen times when his services have been called for under such circumstances. Our advice is often asked for in our office, but the physician in general practice is called upon to officiate.

Generally the physiological eruption of the temporary teeth, their strength, growth, and development is in proportion to the physical health, strength, and development of the child. Some children never have any trouble in the eruption of their teeth, others have irritation with almost all of them.

The permanent teeth can cause no pain in their eruption with the exception of the dens sapienatia, and that only when there is not room for it to take its position in the arch. Accidents happen, anomalies occur, heredity comes in to play its part in the derangement of the dental organs, both in the temporary and permanent sets, when a regular and normal development should be expected. Children are sometimes born with teeth, but these teeth are generally short lived. Teeth that come late in the infant are generally followed by a strong permanent set. Certain peculiarities will be handed down from generation to generation, being shown in the child that resembles the parent having such peculiarities, being often omitted in the child who resembles in features and general characteristics the parent who is free from them. In the teeth this is more generally marked by the absence of one of the temporary or permanent set, usually a lateral incisor, and generally if confined to one side, that side being continued through the whole family.

Much of the condition of the temporary teeth depends on the condition of the mother during gestation. The medicines she takes may leave their impressions on the teeth, particularly is this the case with the various preparations of iron. The muriated tincture is perhaps the most generally given as a tonic, particularly by the old school, and is the most pernicious in its effect on the teeth.

The temporary teeth may be imperfect in their structure, poor in composition, soft and prone to decay; yet they are no indication of what the permanent set will be. It is not usual to see the child of four or five years with the front teeth so badly decayed—eaten away at the neck, face, and sides that there is but little left of them—yet these teeth are often followed by permanent incisors very different in their structure, handsome, regular, and well constituted to resist decay.

While the roots of the temporary teeth are undergoing solution at the approach of the permanent crown, the root vessels and pulp are also absorbed. Should the permanent tooth come directly in the axis of the temporary, that tooth is absorbed from apex to crown. Should the permanent tooth, on the other hand, come a little out of its course so as merely to touch the tooth already in position, it will plow a furrow just in proportion to its contact with that tooth. This would indicate that the contact and pressure of the permanent crown was the cause of the solution, for when the permanent crown does not touch the temporary root no absorption takes place. But while this may be the direct cause, is it the only factor in the case? When the root vessels of a permanent tooth from any cause are absorbed, death of the pulp is an unfailing sequence. This seldom occurs without decomposition following, attended usually by all the essentials necessary to forming an abscess, and generally accompanied by pains of a more or less severe character. Should the roots of a permanent tooth commence to absorb, the sharp edges produced by the absorption act as an irritant, and inflammation and pain are the results. Not so in a tooth of the deciduous set; the root will absorb from apex to crown, pulp and all. While this is going on the portion of the pulp unabsorbed will retain its vitality, and the sharp, lancet-like edges of the root in its process of absorption are tolerated by nature as kindly as the rounded, smooth, natural apex of the root before absorption has commenced.

Whether the absorption of the root of the temporary tooth is the result of direct contact or not, the suggestion is verv strong that there is some agent present with a limited solvent power (perhaps a fluid thrown out) that acts not only as an antiseptic, but as an anæsthetic to prevent the excitement which would naturally follow the presence of two factors usually productive of such results, to wit: an absorbing pulp, and the sharp edges of a partially absorbed root.

It would make this paper too long to enter more fully into this portion of the subject, and the care of the teeth well next be touched on. Uufortunately we seldom see the teeth of our young patients until pain has driven them to us to seek relief from some exposed pulp or aching tooth. Generally the temporary teeth decay first on approximal surfaces of the molars, and this is not noticed by the parents until complaints of pain from the little one attract their attention. When the pain is relieved it should be our duty to call attention to any other teeth in a decaying condition, and they will almost always be found. Those on the opposit side of the ones that have ached will probably be decayed, for as teeth come in pairs, the same influences that produce weakness in one will extend to its mate. Should the decay have advanced so far it cannot be removed by the chisel, file, or disk with safety, it should be removed and the cavity filled with some of the plastic preparations, and such efforts made as will keep the teeth in the mouth in a condition of comfort until they are cast off by the coming permanent set. Should incipient decay be found between the teeth it should at once be removed by liberal separations. Some of our truly progressive and advanced members of the profession advocate anticipating decay by making separations large enough and properly formed to be self cleansing.

Where decay has commenced on the coronal surface nothing can be done but to fill the cavity thus made. The teeth of the temporary set are not sensitive until the nerve is really reached, and even then they are much less so than those of the permanent set in a similar condition. Timidity and fear will often make the child wince, and cry out as if in pain at the removal of the decay but it is seldom the case that real pain exists. From the time the first teeth appear they should be kept scrupulously clean, at first using a soft cloth, then as the child gets older, and the gums more firm, a soft brush should take its place and the necessity of using it be impressed on the child until it will become as much a daily routine as washing the face and hands. This can sometimes be done by a system of rewards to the child every time it remembers to perform this duty until such a course is unnecessary. Every effort should be made to retain the temporary teeth as long as possible. Never remove one unless it is an apparent necessity ; even roots that have lost their crowns are better kept in than removed, if they can be kept comfortable and healthy. The exposed nerves of the temporary teeth are easily devitalized by one or two applications of creosote or other escharotic. Powerful devitalizers should never be used on the teeth of a child.

It is too much the custom with many dentists, when a child presents itself with an aching molar to immediately remove it, not caring whether it hurts or not, and no one but a child who has passed through the ordeal knows how much it does hurt. Whether this removal retards the growth of the maxillary or not, the practice is a bad one if for no other reason than it teaches the child to dread the dental office from the lasting impression made by the pain received, and in this way (the dread of the dentist) comes the neglect of the teeth. Many a patient has traced back to the first visit to a dental office and the shock received the cause of the loss of many of the dental members.

The removal of the temporary teeth that have not been absorbed at the roots thus causing the permanent teeth to be thrown out of the arch, is a question that requires much judgment, as much harm may be caused by their retention as by their removal at an improper time, all the surroundings should be well balanced before we operate.

Kindness, gentleness, and winning the confidence of a child by never deceiving it, go a long way in the ultimate saving of teeth. Never tell a child it will not hurt when you know it will. Give children short sittings and do not tire them.

As conservers of the dental organs it is our duty to try and teach our patients from the time they come into our hands, and we should try and save them all possible pain. We cannot begin too early to educate them. An impression made on a child is more lasting than one made on an adult.

Much has been said about the necessary education of parents that they may properly appreciate the value of their children's teeth, and see that they have the proper care at an early age. It we educate the child we have educated the future parent and further education at our hands will be unnecessary.

